# A fluorescent metal-sensor study provides evidence for iron transport by transcytosis in the intestinal epithelial cells

**DOI:** 10.3164/jcbn.17-74

**Published:** 2017-12-12

**Authors:** Yuxiang Ma, Yasumasa Okazaki, Jonathan Glass

**Affiliations:** 1 Feist-Weiller Cancer Center and the Department of Medicine, Louisiana State University Health Sciences Center-Shreveport, 1501 Kings Highway, Shreveport, LA 71130, USA

**Keywords:** iron absorption, calcein, iron transport, Caco-2, hemin

## Abstract

Iron transport across the intestinal epithelium is facilitated by the divalent metal transporter 1 (DMT1) on the brush border membrane (BBM). The fluorescent metal sensor calcein, which is hydrophilic, membrane-impermeable and quenched by chelation with iron, was used to test our hypothesis that intestinal iron absorption is through the endocytic processes and is involved in a pathway where BBM-derived vesicles fuse with basolateral membrane (BLM)-derived vesicles. To monitor the flux of iron via transcytosis, Caco-2 cells were employed as a polarized cell layer in Transwell chambers. When calcein was added to the basal chamber along with apo-transferrin (apo-Tf), calcein rapidly underwent endocytosis and co-localized with apo-Tf. Calcein was quenched by adding an iron-ascorbate complex and then restored by adding 2,2'-dipyridyl into the apical chamber. These results were confirmed by live-cell imaging. When hemin from the apical surface and calcein from the basal chamber were added to the Caco-2 cells, internalization of DMT1 and quenching of calcein were not observed until 2 h later. These results indicated that absorbed hemin required processing before hemin-derived iron was available to BLM-derived vesicles. These studies suggest that iron is transported in Caco-2 cells by transcytosis with apical-derived vesicles that are fused to BLM-derived vesicles.

## Introduction

Iron is required by various ferroproteins and heme-containing proteins that are essential in a wide variety of species for cell growth and survival. These proteins are involved in a myriad of cell functions including DNA synthesis, oxygen transport, and cell respiration. For the purpose of iron utilization and storage, all living cells have available iron, which is stored as ferritin and in an intracellular pool called the labile iron pool (LIP). This LIP, defined as a pool of chelatable and redox-active iron, is transitory and serves as a crossroad for cellular iron metabolism.^(1)^ Principally, iron stores are maintained by regulating iron absorption from the duodenum. Iron deficiency causes microcytic anemia and slow-growing, on the other hand, iron overload generates reactive oxygen species that cause oxidative stress and cellular injury, subsequently, cancer in the body.^(2)^ Many patients of hemochromatosis which is characterized by iron overload in liver, heart, pancreas and reproductive organs, suffer liver dysfuction and died from the liver cirrhosis and hepatocellular carcinoma. An iron chelator could prevented the carcinogenesis in liver, which plays a central role in iron storage.^(3,4)^ Thus, iron-storage is tightly regulated in the duodenum. Dietary ferric iron is reduced to ferrous iron by duodenal cytochrome b, which is located on the brush border membrane (BBM). This ferrous iron is transported across the BBM of the duodenum via the divalent metal transporter 1 (DMT1) (SLC11A2).^(5–14)^ DMT1 is a co-transporter of protons and divalent metals with an optimum pH of approximately 5.5 for iron transport.^(7,12,15)^ Ferrous iron was thought to be transported to the basolateral membrane (BLM) and to exit across the BLM from the enterocyte via ferroportin (FPN) (SLC40A1).^(9,10,16)^ Recently, poly(rC)-binding protein 1 and 2 (PCBP1, 2) were shown to regulate the transfer of iron by ferritin or FPN.^(17,18)^ We and others have observed that in the intestinal epithelial cells iron feeding triggered the internalization of DMT1 from the BBM into vesicles within the subapical cytosolic compartment,^(14)^ and hepaestin (Heph) and FPN migrated from the sub-apical compartment to the BLM.^(16,19,20)^ It is not unclear if the uptake of iron is dependent on a regulatory mechanism via transcytosis of vesicles derived from the apical BBM and vesicles derived from the BLM.

In this study, we employed green fluorescent calcein, which possesses hydrophilic and membrane-impermeable properties. This compound is able to be taken into cells by endocytosis. The fluorescence is quenched by iron, hence, making it possible to determine the coefficients of physiological iron influx into the cytosolic and mitochondrial labile iron pools.^(1,21)^ Caco-2 cells, grown as a polarized cell layer in Transwell chambers as a model system to study iron flux, were combined with calcein. The established experimental model operated as follows: the apical chamber bath acts as the BBM of the enterocytes and the basal chamber bath acts as the BLM.^(8,22,23)^ Using this model, calcein and apo-transferrin (Tf) were added into the basal chamber. Iron and 2,2'-dipyridyl (DPD), which is a membrane-permeable iron chelator, were placed into the apical chamber. To further investigate the mechanism of kinetic transcytosis, live-cell imaging was performed with calcein and dextran.

Previous studies of Caco-2 monolayers demonstrated that adding hemin to the apical chamber allowed hemin to be transported into the basal chamber without degradation, suggesting the prevention of iron overload in the cells.^(24)^ Hence, the impact of iron that is released from hemin feeding on the labile iron pools still remains unclear. We also examined the effect of hemin (ferric protoporphyrin IX) in this model.

## Materials and Methods

### Materials

TOPRO-3, ProLong Antifade kit, calcein, Alexa 546-conjugated dextran (M.W. 10,000), Alexa 594-conjugated-Tf, and non-essential amino acid solution (10 mM) were from Invitrogen (Carlsbad, CA). Fetal bovine serum (FBS) was from Atlanta Biologicals (Lawrenceville, GA). DMEM was from Mediatech (Herndon, VA) or Sigma Aldrich (St. Louis, MO). Paraformaldehyde was from Fisher Scientific (Pittsburgh, PA). HEPES, Antibiotics, Chelex resin, ascorbate, ferric chloride, hemin and 2,2'-dipyridyl (DPD) were from Sigma Aldrich. All other chemicals of analytical grade were from Sigma Aldrich. Anti-DMT1 antibody (iron-responsive element, IRE) was described previously.^(14)^ The Caco-2 cell line (HTB-37) was from American Type Culture Collection (Rockville, MD). The Millicell electrical resistance system and Millicell 6-well cell culture inserts with 0.4 µm pore size membranes were from Millipore (Bedford, MA). Transwell clear inserts for 24-well plates were from Corning (Lowell, MA). The collagen solution (rat tail Type I) was from Boehringer-Manheim (Manheim, Germany) and Becton Dickinson (San Diego, CA). DRAQ5 was from AXXOLA (San Diego, CA). The Bio-Rad Radiance 2000 Laser scanning microscope and Lasersharp 2000 were from Bio-Rad (Hercules, CA). The LSM 510 META Confocal microscope was from Carl-Zeiss (Thornwood, NY). Glass-bottom dishes were from Mat-Tek (Ashland, MA).

### Cell culture

Caco-2 (HTB 37) cell line was grown and maintained in DMEM supplemented with 10% FBS, non-essential amino acid solution (0.1 mM) and antibiotics (100 U/ml penicillin-G and 100 U/ml streptomycin). The collagen film was applied to the membrane by adding the collagen solution. Preparation of the Caco-2 monolayer was described previously.^(8)^ In brief, Caco-2 cells were seeded onto the membrane and cultured to form a monolayer. Transepithelial electrical resistance (TEER) was measured in this monolayer with the Millicell electrical resistance system. Confocal microscopy experiments were performed only after the TEER had risen to a level indicating the formation of an intact monolayer (TEER at least 400 Ω/cm^2^). Typically, the cell monolayers were used after 12–14 days.

### Iron transport study with calcein and Alexa 594-conjugated Tf

Before each experiment, the cell monolayer was incubated with serum free DMEM overnight. After being washed three times with HEPES buffered isotonic solution (193 mM HEPES, 130 mM NaCl, 10 mM KCl, 1 mM CaCl_2_, 1 mM MgSO_4_, pH 7.4), Caco-2 monolayer was used. Calcein (200 µM) and Alexa 594-conjugated Tf (2.5 µM) were added to the basal chamber and incubated for 20 min and then washed three times with HEPES buffered isotonic solution. After extensive washing with phosphate-buffer saline (PBS), iron-ascorbate complex (5 µM Fe, 500 µM ascorbate) were placed into the upper chamber to induce endocytosis, which was mediated by DMT1 and incubated for 20 min. In some experiments, after adding Fe-ascorbate, DPD (200 µM) was added into the upper chamber and incubated for 5 min. After the indicated time, the monolayer was fixed in paraformaldehyde (2%) and mounted with ProLong Antifade kit after extensive washing with PBS. Fluorescent images were obtained by a confocal microscope from Bio-Rad.

### Live cell imaging by laser scanning confocal microscopy

Before each experiment, the cell monolayer was incubated with serum-free DMEM overnight. In addition, the monolayer was washed three times with HEPES buffered isotonic solution, then incubated for 30 min with DRAQ 5 (10 µM) in a CO_2_ incubator. After the Millicell 6-well cell culture insert with the Caco-2 cell monolayer was transferred onto the glass-bottom dish, we observed the living cells under a laser scanning confocal microscope (LSM 510 META) with temperature control. Images were collected using a 63× dipping objective. Calcein (200 µM) and Alexa 546-conjugated dextran (M.W. 10,000) (2.5 µM) were added into the basal chamber. After 1, 5, 10 and 20 min, kinetic images were taken. 20 min later, this chamber was extensively washed three times with HEPES-buffered isotonic solution. After 30 min of incubation on a temperature-controlled stage, we added the iron-ascorbate complex (5 µM Fe, 500 µM ascorbate) into the apical chamber; then we took images at the 1-, 5- and 10-min time points. After this kinetic study, we washed the chamber three times, as in the previous step. Then, we added DPD (200 µM) into the apical chamber. We took images at the 1-, 5- and 10-min time points. In some experiments, we mechanically loosened the monolayers, which scratched the membrane causing the monolayer of Caco-2 to float in the buffer, to investigate the functional role of basolateral tight junction.

### Labile iron pools studied with calcein, after hemin feeding

Caco-2 was described above. After the administration of calcein and extensive washing, hemin (50 µM) was added into the upper chamber. After the indicated time, the monolayer was fixed and fluorescent images were examined with a confocal microscope.

### Immunohistochemical staining

Caco-2 grown on coverslips were fixed with 2% paraformaldehyde for 10 min at room temperature (RT). After extensive washing with PBS for 15 min, these sections were permeabilized with Triton X-100 (0.1%, v/v) and blocked with blocking solution (5% BSA and 1% goat serum in PBS) for 30 min at RT. Subsequently, these sections were incubated with anti-DMT1 (IRE) serum (1:500) for 2 h at RT. Following extensive washing with PBS, the samples were incubated with Alexa 488-labeled goat anti-rabbit IgG (1:500) and TOPRO-3 (1:1,000) for 1 h at RT. Samples were mounted with a ProLong Antifade kit after extensive washing with PBS. Fluorescent images were obtained with a confocal microscope from Bio-Rad.

### Statistics

Fluorescent signal intensities and areas were calculated using MetaMorph. Where indicated, statistical comparisons were made with an unpaired Student’s *t* test.

## Results

### Calcein highlighted basolateral vesicles and fused with apical ferrous iron

**Fig. 1:**
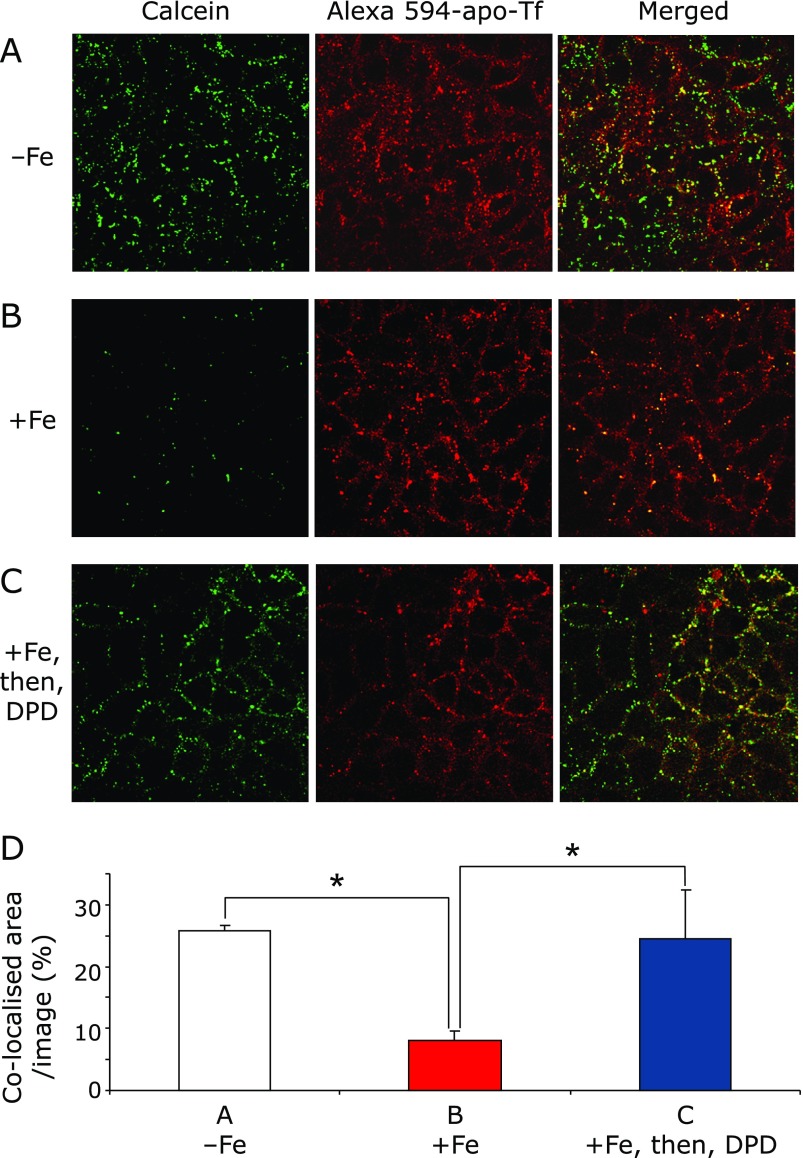
Co-localization of the metallosensor calcein and apo-transferrin (Tf) were examined in different iron conditions by confocal microscopy. (A) Observation without iron feeding. After calcein and apo-Tf were added into the basal chamber and incubated for 20 min, the Caco-2 monolayer was extensively washed and fixed. (B) Observation with iron feeding. After extensive washing, iron-ascorbate complex (5 µM Fe, 500 µM ascorbate) was added into the apical chamber and incubated for 20 min with the Caco-2 monolayer. (C) Observation of 2,2'-dipyridyl (DPD) addition after iron feeding. After iron feeding, DPD (200 µM) was added into the apical chamber and incubated for 5 min. Shown images are representative of the fluorescent probes. (D) Digital analysis of fluorescent signals. Images, which were collected in (A), (B) and (C) experimental conditions, were analyzed by counting the signal intensity and area in digital pixels by MetaMorph. These analyses are expressed in co-localized area/image (%) mean ± SD for at least three independent experiments. * indicates a significant difference (*p*<0.05).

Calcein rapidly underwent endocytosis and co-localized with apo-Tf, which was labeled with Alexa 594 in the sub-apical cytoplasm with a coefficient of co-localization of 26.8 ± 3.6% (Fig. 1A and D). The green signal from calcein was quenched by adding iron ascorbate complex (5 µM Fe, 500 µM ascorbate) into the apical chamber (Fig. 1B and D). This result clearly demonstrated that vesicles from the BLM and BBM containing ferrous iron had fused in the cytoplasm. This ferrous iron was reduced by DPD, which can decrease LIP. After adding DPD to the apical chamber, the green signal from calcein and the co-localization ratio were restored (Fig. 1C and D). These results demonstrated that calcein and apo-Tf were localized in close compartment.

### Dynamic vesicles visualized by calcein in live cell imaging

**Fig. 2:**
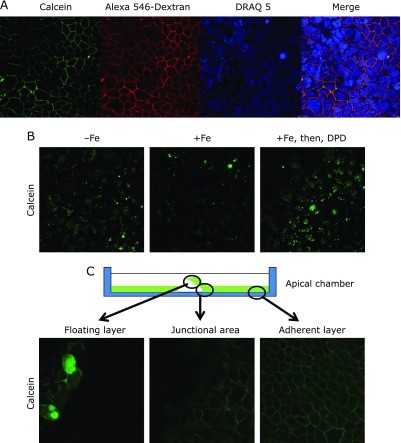
Live cell imaging of the Caco-2 monolayer. (A) Uptake of fluorescent probes by endocytosis from basolateral membrane. Nuclei of the Caco-2 monolayer were stained with DRAQ 5 (10 µM) in a CO_2_ incubator for 30 min. Calcein (200 µM) and Alexa 546-conjugated dextran (M.W. 10,000) (2.5 µM) were added into the basal chamber. After 5 min, confocal images were collected. (B) Dynamic change of calcein after iron feeding and 2,2'-dipyridyl (DPD) addition. In images without Fe, cytoplasmic signals were visualized after extensive washing with HEPES-buffered isotonic solution. In images with Fe, addition of the iron-ascorbate complex (5 µM Fe, 500 µM ascorbate) quenched the fluorescent signals of calcein. In images with Fe, then, DPD (200 µM), after extensive washing with HEPES-buffered isotonic solution, DPD (200 µM) was added into the apical chamber. The green signals derived from calcein were restored. (C) Mechanically loosened monolayer during the uptake of calcein. We scratched the monolayer to break the tight junctions and float the monolayer in the buffer. In the floating layer, a few Caco-2 cells contained calcein probe in the entire cytoplasm; however, the remainder of the Caco-2 monolayer demonstrated basolateral localization. Both the junctional area and adherent layer demonstrated basolateral localization.

Calcein and dextran, which are markers of endocytosis, were taken up by the BLM (Fig. 2A). These signals were observed, even 1 min after adding dye into the basal chamber. Under these conditions, we could detect fluorescent signals with low laser-emission power. After washing and waiting for the cytoplasmic spread of calcein, we observed cytoplasmic calcein signals with higher laser-emission power (Fig. 2B). Adding iron ascorbate complex (5 µM Fe, 500 µM ascorbate) into the apical chamber quenched the fluorescent green signals (Fig. 2B), indicating that the vesicles from the apical and basolateral compartments fused and formed a new vesicle to transmit iron. After administration of DPD into the apical chamber, green signals were restored (Fig. 2B). Only high laser-emission, leading to photobleaching, enabled us to detect these signals. Our initial attempt to monitor the kinetic variation of calcein was not a quantitative result. However, the conventional method and the real-time monitoring agreed in the localization of the fluorescent probe and the response to iron feeding. We observed the same localization of fluorescent probes in the intestinal monolayer model, using T84 cell lines (alternative polarized intestinal model) (data not shown). To investigate the physiological role of basolateral tight junctions in polarized Caco-2 cells, we tried to loosen the monolayer mechanically. We observed basolateral localization in the adherent layer and junctional area (Fig. 2C). In the floating layer, although some Caco-2 monolayer showed basolateral localization, a few Caco-2 cells in the same setting contained calcein throughout the cytoplasm (Fig. 2C).

### Immunofluorescent staining of DMT1 (IRE) after feeding with hemin

**Fig. 3:**
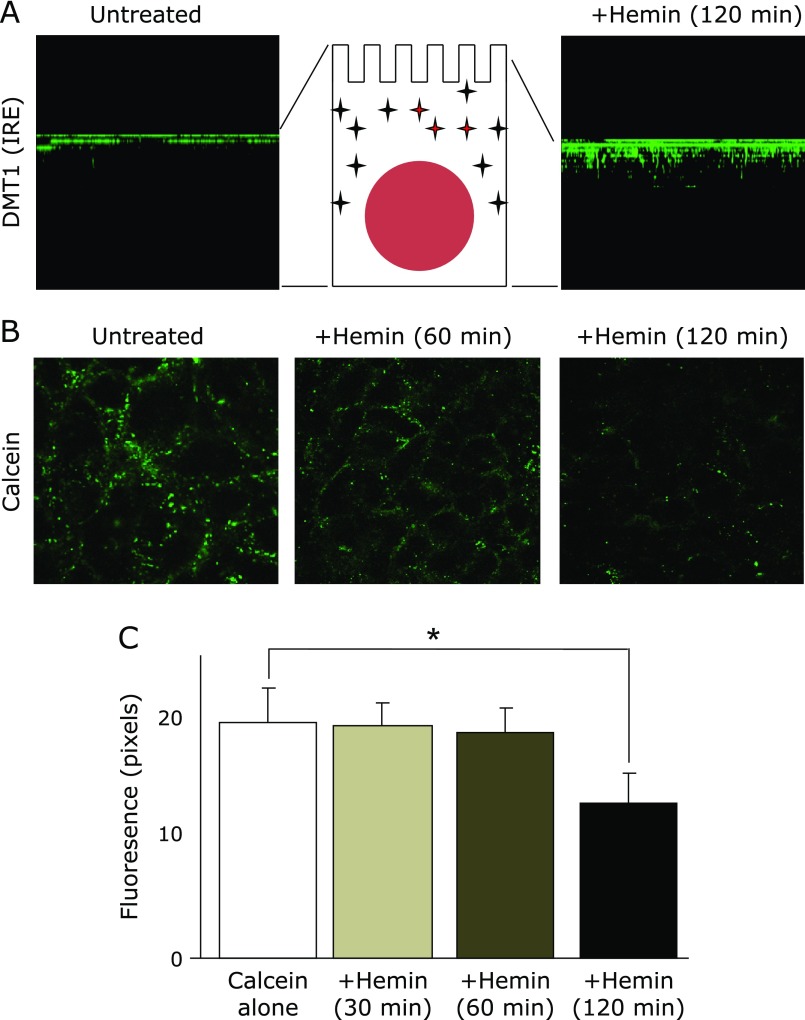
Impact of hemin feeding on DMT1 (IRE) internalization and labile iron pools. (A) Immunofluorescent staining of DMT1 (IRE) in the Caco-2 monolayer. (Left) Before feeding, DMT1 (IRE) was localized in the BBM. (Right) After feeding with hemin (50 µM) for 2 h, DMT1 (IRE) migrated into the subapical compartment. These images were reconstructed to be viewed from a lateral perspective by taking sections along the *z*-axis with a step size of 2 µm to give ~12 sections per imaged field. (B) Determination of the labile iron pools by calcein after hemin feeding. After feeding with hemin (50 µM), Caco-2 monolayers were stained with calcein (200 µM). Quenching of these fluorescences were observed 120 min later but were not significant 30 and 60 min later. (C) Digital analysis of fluorescent signals. Images, which were collected after hemin feeding, were analyzed for signal intensity and area in digital pixels by MetaMorph. These analyses were expressed in fluorescence mean ± SD for at least three independent experiments. * indicates a significant difference (*p*<0.05).

Internalization of DMT1 (IRE) was observed in polarized Caco-2 cells after being fed hemin for 2 h (Fig. 3A). These images clarified that DMT1 (IRE) migrated into the sub-apical compartment after the hemin feeding. This result implied that hemin-induced internalization of DMT1 (IRE) required at least 2 h for the degradation of hemin into carbon monoxide, biliverdin and ferrous iron.

### Determination of the labile iron pools by calcein after hemin feeding

Based on these findings, we performed a fluorescent study to see the effects on LIP imposed by hemin. We observed that the green signal was significantly quenched 120 min later, but not at 30 and 60 min (Fig. 3B and C). These results suggested that Caco-2 cells needed time to turn hemin into the available ferrous iron.

## Discussion

Iron is essential for the cellular growth and survival of all living cells. This pivotal element is absorbed from dietary sources by the gut. The duodenum is the critical site for iron absorption that maintains iron homeostasis in mammals. In the duodenum, DMT1 (IRE) plays a central role in iron transport across the duodenal BBM.^(5–14)^ The iron was stored in enterocytes and exited across the duodenal BLM, which is mediated by hephaestin and FPN. This iron flux was thought to be triggered by the LIP in enterocytes. Here, we employed a polarized Caco-2 model using a Transwell chamber with the metallosensor calcein, which helps visualize the LIP to investigate transcytosis in the intestinal epithelial cells. In this study, we provide evidence that BBM-derived vesicles, which contained iron, fused with BLM-derived vesicles, which contained calcein. This quenching was restored by the addition of DPD in the apical chamber. These studies are suggesting that iron is transported in Caco-2 cells by transcytosis with apical-derived vesicles fusing to BLM-derived vesicles.

To determine the kinetic movement of vesicles in iron absorption, we observed Caco-2 monolayers with a live cell imaging technique. Calcein was quenched by iron feeding and restored with the addition of DPD. This Transwell model was too distant from the laser and detector, so it was necessary to discharge the high-emission laser power, causing photobleaching of calcein (data not shown). These results were consistent with the conventional method, described above, although this method was not sufficiently quantitative. Previously, live cell imaging of calcein was effective at determining the coefficients of physiological iron influx and stained the whole cytoplasm in K562, HepG2, H9C2 and J774 cell lines, which do not have polarization.^(21,25)^ The other colorimetric and fluorescent dyes for the detection of iron, such as RhoNox-1, are summarized.^(26)^ The application of RhoNox-1 in clinical samples, including maternal samples has been carried to demonstrate the biological significance of ferrous iron.^(27)^

We speculated that tight junctions between the polarized intestinal epithelial cells play essential roles in endocytosis from the BLM. To challenge this hypothesis, we used mechanically loosened monolayers to damage the tight junctions. In this model, we observed staining of the whole cytoplasm in a few Caco-2 cells in the floating monolayer, however, BLM localized in the remainder of the Caco-2 cells (Fig. 2C). This suggested that tight junctions between polarized cells were important for endocytosis and for the delivery of the vesicles to specific compartments.

To investigate the intestinal iron absorption from dietary iron resources, we observed the effect of hemin feeding on the LIP in enterocytes. We observed the internalization of DMT1 (IRE) after hemin feeding at 2 h later (Fig. 3A). We confirmed that calcein did not spontaneously exit from the cytoplasm for 240 min in this model (data not shown). The quenching of calcein was observed at 120 min later but was not significant at 30 or 60 min later, which was not enough time for hemin-derived iron to quench the fluorescence. This suggested that there was a time-lag before hemin-derived iron could recruit DMT1 (IRE) and enter into the LIP for further bioavailability. The vesicles containing non-heme iron would have fused with BLM-derived vesicles, leading to exit iron from the BLM. The system transporting iron-containing vesicles from the BBM to BLM still remains to be elucidated. To find a clue to understand the systematic endocytosis of DMT1, proteins that interact with DMT1 have been explored. The interaction of the N-terminus of DMT1 with PCBP2, which also binds to FPN and heme oxygenase-1, is shown.^(18,28)^ The interaction of the C-terminus of DMT1 (IRE) with PAP7 was shown in PC-12, Caco-2, K562 cells and in the duodenal tissue of rats.^(29,30)^ Although PAP7 and PCBP2 may affect vesicular travel in the cytosol, detailed mechanisms are still unknown.

DMT1 is also known as a metal transporter for Cadmium^2+^, Cobalt^2+^, Manganase^2+^ and Zinc^2+^. An intestine-specific ablation of DMT1 mouse model disclosed that DMT1 was not critical for copper, manganese and zinc transport.^(11)^ Mutations in human DMT1 cause the onset of microcytic anemia and hepatic iron overload, thus, alternative pathways of iron uptake in the liver were hypothesized to be involved, including alternate activation of heme-iron absorption and/or the activation of residual DMT1 isoforms.^(12)^ Zrt-like Irt-like protein 14 (ZIP14) was shown to be alternative transporter in cellular uptake of iron.^(31)^ However, the optimal condition of ZIP14 was pH 7.5 in contrast to DMT1 (pH 5.5).^(11)^ The activity of ZIP14 as an iron transporter is thought to be suppressed in the duodenum under acidic conditions.

In conclusion, we have provided evidence that two forms of dietary iron resources were transported by the fusing of vesicles from the BBM and BLM in the Caco-2 cells. This study highlighted the concept that iron is absorbed by transcytosis in polarized enterocytes.

## Author Contributions

J. G. designed this study. Y. M. and Y. O. acquired the data. J. G., Y. M. and Y. O. analyzed and interpreted the data. Y. O. drafted the manuscript and J. G. revised the manuscript.

## Abbreviations

BBM: brush border membrane
BLM: basolateral membrane
DMT1: divalent metal transporter 1
DPD: 2,2'-dipyridyl
FBS: fetal bovine serum
FPN: ferroportin
IRE: iron responsive element
LIP: labile iron pool
PCBP2: poly(rC)-binding protein 2
RT: room temperature
TEER: transepithelial electrical resistance
Tf: transferrin
